# Serum and Skin Carotenoid Levels in Older Adults with and Without Metabolic Syndrome: A Cross-Sectional Study

**DOI:** 10.3390/nu17193049

**Published:** 2025-09-24

**Authors:** Susan Veldheer, Dongxiao Sun, Polly S. Montgomery, Ming Wang, Xue Wu, Menglu Liang, Susan George, Andrew W. Gardner

**Affiliations:** 1 Department of Family and Community Medicine, Penn State College of Medicine, Hershey, PA 17033, USA; 2 Department of Public Health Sciences, Penn State College of Medicine, Hershey, PA 17033, USA; 3 Department of Pharmacology, Mass Spectrometry Core Facilities, Penn State College of Medicine, Hershey, PA 17033, USA; dsun@pennstatehealth.psu.edu; 4 Department of Physical Medicine & Rehabilitation, Penn State College of Medicine, Hershey, PA 17033, USAandrew-gardner@ouhsc.edu (A.W.G.); 5 Department of Population and Quantitative Health Sciences, School of Medicine, Case Western Reserve University, Cleveland, OH 44106, USA; 6 Department of Epidemiology and Biostatistics, School of Public Health, University of Maryland, College Park, MD 20742, USA; 7 Department of Biochemistry and Molecular Biology, Penn State College of Medicine, Hershey, PA 17033, USA; 8 Department of Medicine/Cardiovascular Section, University of Oklahoma Health Sciences Center, Oklahoma City, OK 73104, USA

**Keywords:** serum carotenoids, skin carotenoids, metabolic syndrome, cardiovascular disease, fruit and vegetable intake

## Abstract

**Introduction:** Metabolic syndrome (MetS), a clustering of cardiovascular disease (CVD) risk factors, is associated with increased mortality. Fruit and vegetable (FV) intake is inversely associated with CVD risk, and carotenoids, bioactive compounds found in brightly colored FVs, can be measured in serum and skin as biomarkers of intake. While serum and skin carotenoids are correlated in healthy populations, this relationship is not well understood in older adults with MetS, who may have altered carotenoid absorption or metabolism. **Methods:** In this cross-sectional study, adults aged 55+ were assessed for serum carotenoid concentrations, pressure-mediated reflection spectroscopy (RS) skin carotenoid scores, self-reported FV intake, sociodemographic characteristics, and comorbidities. MetS status was determined using the National Cholesterol Education Program Adult Treatment Panel III criteria (77 with MetS, 63 without). Linear regression models evaluated group differences in carotenoid levels. Associations between serum and skin carotenoids were examined using Spearman correlation and multivariable regression. **Results:** Participants with MetS had significantly lower serum alpha-carotene (52%), beta-carotene (39%), and total carotenoids (22%) than those without MetS (all *p* < 0.002). Differences remained after adjustment for sociodemographic and health-related factors. No significant group differences were found for lycopene, lutein, cryptoxanthin, or skin carotenoid scores. Total serum carotenoids were positively correlated with skin scores (r = 0.58, *p* < 0.001), and this association persisted in adjusted models. **Conclusions:** Older adults with MetS had lower serum carotenoid levels, primarily due to alpha- and beta-carotene. This serum–skin correlation supports RS-based skin measurement as a practical, non-invasive assessment of carotenoid status.

## 1. Introduction

Metabolic syndrome (MetS) is a cluster of cardiovascular disease (CVD) risk factors which include abdominal obesity, hyperglycemia, dyslipidemia, and hypertension [1]. MetS is highly prevalent affecting 25% of the global population [2] and 38% of the US population [3], with estimates expected to increase due to corresponding increases in obesity and diabetes [4,5]. MetS is clinically significant because it is associated with increased risk of all-cause mortality [6,7,8], cardiovascular mortality [6,7,8], CVD [6], major cardiovascular events [8], and diabetes [1]. Additionally, MetS is associated with a 60% increase in health care costs [9].

There is a well-documented inverse dose relationship between CVD mortality and fruit and vegetable (FV) intake [10], with estimates suggesting a 4% reduction in CVD mortality for each additional serving of FV consumed up to 5 servings per day [11]. Thus, increasing FV intake is a critical goal of clinical interventions targeting CVD prevention, particularly among older adults. However, evaluating the effectiveness of such interventions is complicated by challenges with accurately measuring dietary intake in clinical settings. Gold-standard dietary assessments, such as 24-h recalls or Food Frequency Questionnaires (FFQs), can provide a detailed description of individual dietary intake but are resource intensive, subject to recall bias, and often impractical for routine clinical settings. These challenges may be further compounded in older adults who may experience cognitive or visual impairments that limit accurate self-reporting [12].

Validated, measured markers of FV intake such as serum or optical skin carotenoid levels may be an alternative option. Carotenoids are obtained from the diet through brightly colored FV, with the majority of biologically measurable carotenoids coming from five compounds: α-carotene, β-carotene, lutein, lycopene, and cryptoxanthin [13,14]. Serum carotenoids are highly correlated with dietary intake of FV and have a relatively stable half-life from 2 to 10 weeks, which allows them to serve as a measure of longitudinal change in FV intake [15,16,17,18]. However, serum carotenoid collection requires venipuncture, processing, sample storage (in −80 freezers), and specialized laboratory analysis; procedures that can be costly and logistically challenging in community or clinical settings.

In response, non-invasive optical methods for measuring carotenoid levels in skin have emerged, including Raman Resonance Spectroscopy (RRS) and pressure-mediated reflection spectroscopy (RS) [19,20]. These methods eliminate the need for venipuncture, sample processing, sample storage or analysis, making them especially well-suited for older adult populations and real-world clinical settings. The present study used the RS method obtained via the Veggie Meter, a portable, research-grade device that enables rapid, non-invasive assessment of carotenoid status in clinical and community environments [19,21].

Several studies have found that blood carotenoid levels are approximately 8–15% lower in adults with MetS as compared to those without [22,23,24,25]. However, despite the high burden of MetS among older adults, few studies of skin and serum carotenoids have focused on this population when examining the relationship between carotenoids and metabolic health [26,27]. Similarly, validation studies of skin and serum carotenoids have primarily been conducted in healthy children [28] and middle-aged adults [19] leaving a critical knowledge gap regarding their validity in older adults. Therefore, the primary aim of this study was to compare serum carotenoid concentrations and RS skin carotenoid scores in older US adults with and without MetS. A secondary aim was to determine whether total serum carotenoid concentrations were associated with skin carotenoid scores.

## 2. Materials and Methods

The present analysis was conducted using data from a cross-sectional study designed to examine the inter-relationships between walking ability, vascular biomarkers, physical activity, and dietary habits in adults aged 55 years and older. In the present study, we focused on comparing serum carotenoid concentrations and skin carotenoid scores in participants with and without metabolic syndrome (MetS) and on examining the correlation between these measures. All data for this report were collected during a single study visit following standardized protocols.

### 2.1. Participants

*Human Ethics and Consent to Participate.* This study was approved by the Penn State College of Medicine institutional review board (IRB). Written informed consent was obtained from each participant before the study. Registration for a clinical trial number was not applicable.

*Recruitment.* Participants were recruited using IRB-approved advertisements. Interested participants were subsequently evaluated at the Penn State Clinical and Translational Science Institute (CTSI), and were screened on inclusion and exclusion criteria that have been used previously [29], and which are listed below.

*Inclusion and Exclusion Criteria.* Participants were included in the study if they met the following criteria: (a) age ≥ 55 years, and (b) ambulatory without the need of an assistive device. Participants were excluded for the following conditions: (a) age < 55 years, (b) non-ambulatory, (c) neurological diseases (including but not limited to Parkinson’s disease, Alzheimer’s disease, multiple sclerosis, amyotrophic lateral sclerosis), (d) active cancer, and (e) stage 5 chronic kidney disease (end stage), as defined by an estimated glomerular filtration rate < 15 mL/min per 1.73 m [30].

### 2.2. Tests and Measurements

*Medical Screening.* Participants were fasted when they arrived at the CTSI in the morning and were allowed to take their usual medications. Research staff administered questionnaires and recorded socio-demographic information from the participants onto research data forms. Blood pressure was obtained using a Dinamap monitor (WelchAllyn Spot Vital Signs, Skaneateles Falls, NY, USA). Height was obtained using a stadiometer (SECA 213, Hamburg, Germany) after the participant had removed their shoes. Minimal waist circumference was measured using a flexible, non-stretchable tape measure while participants stood. The tape was wrapped around the minimal waist parallel to the floor. Ankle/brachial index measurements were obtained according to standard guidelines [31]. Once the clinical measurements were complete, participants had two blood samples drawn. One sample was analyzed for a complete metabolic panel, lipid panel, and insulin by the hospital central lab. The remaining sample was processed in a refrigerated centrifuge, aliquoted, and stored in a −80 freezer awaiting batch analysis of serum carotenoids.

Participants underwent a medical history and physical examination conducted by study physicians, in which comorbid conditions, cardiovascular risk factors, and current medications were recorded. Based on the results of this battery of baseline assessments, participants were coded on cardiovascular risk factors according to definitions for hypertension, dyslipidemia, diabetes mellitus, obesity (BMI ≥ 30), and abdominal obesity (cut points listed below) [32]. Additionally, a history of coronary artery disease, cerebrovascular disease, peripheral artery disease, and chronic kidney disease [30] were obtained during a medical history by study physicians and coded according to standard definitions, as previously described [33]. A history and symptoms of arthritis, and chronic obstructive pulmonary disease were also recorded. The physical examination concluded with the assessment of peripheral neuropathy, as a 10 g Semmes-Weinstein monofilament evaluation was performed at 10 sites on each foot [34,35], and a vibration perception test was performed using a C 128 Hz tuning fork applied to the big toe, first metatarsal joint, ankle, and knee [36]. Peripheral neuropathy was recorded if the participants did not correctly perceive the application of the monofilament or the disappearance of the vibration of the tuning fork at the various locations.

*Body-Composition Assessment.* Body fat percentage, body weight, and body mass index were obtained using a model D1000-3 eight-electrode bio-electric impedance machine (Rice Lake Weighing Systems, Rice Lake, WI, USA) while participants stood barefoot on two stainless-steel rectangular foot-pad electrodes on the base of the machine and held hand grip electrodes [37,38]. Participants emptied their pockets prior to the measurement, and were wearing comfortable clothing. The body composition results were obtained immediately after the test from a print out from the bio-electric impedance machine.

*Serum Carotenoid Concentration.* Serum carotenoids were assessed via High-Performance Liquid Chromatography (HPLC) coupled with Mass Spectrometry (MS), which included a Sciex QTRAP 6500+ MS coupled with a Sciex EXion HPLC separation system [39,40,41,42,43]. A YMC carotenoid column (250 × 4.6 mm, S-5 μm, YMC separation Technology, Kyoto, Japan) was used to separate the 5 carotenoids of interest (α-carotene, β-carotene, lutein, lycopene and cryptoxanthin) from other isomers and impurities. The gradient elution was conducted using a flow rate of 0.8 mL/min with gradient elution, with mobile phase A (10 mM ammonium acetate in methanol with 0.1% formic acid) and mobile phase B (10 mM ammonium acetate in MTBE: MeOH (80:20) with 0.1% formic acid). The autosampler was kept at 4 °C, and the column temperature was maintained at 40 °C. ^13^C_4_-beta-carotene was used as internal standard, and QTRAP 6500+ MS was equipped with an atmospheric pressure chemical ionization (APCI) probe operated in positive mode. Given the nonpolar nature of carotenoids APCI was employed for efficient ionization and sensitivity of quantification [42,43]. The decluster potential (DP) was 70–100 V; the entrance potential (EP) was 10 V; the collision energy (CE) was 18–28 V; the collision cell exit potential (CXP) was 12.8–18 V. The curtain gas (CUR) was 35 L/h, the collision gas (CAD) was high. The ionspray voltage was 5500 V, and the temperature was 400 °C, with the nebulization gas at 3, and gas 1 at 40 L/h, gas 2 at 0 L/h. The multiple reaction monitoring mode (MRM) was used to analyze and quantify carotenoids with the transitions of *m*/*z* 537 > 413 for α-carotene, β-carotene, lycopene, *m*/*z* 553.5 > 461 for cryptoxanthin, *m*/*z* 551 > 429 for lutein, and *m*/*z* 541.5 > 417 for ^13^C_4_-β-carotene. All peaks were integrated and quantified by Sciex OS 1.5 software. This study focused exclusively on targeted quantification of α-carotene, β-carotene, lutein, lycopene, and cryptoxanthin since these 5 compounds represent >85% of total dietary intake [13,14]. No untargeted metabolomics analyses were performed.

*Skin Carotenoid Score.* Skin carotenoid scores were obtained on a sub-sample of participants using the pressure-mediated reflection spectroscopy (RS) method (Veggie Meter^®^, Longevity Link Corporation, Salt Lake City, UT, USA) [20]. The sub-sample occurred because, shortly after study initiation, investigators determined that incorporating skin carotenoid measurements was both feasible and scientifically valuable. However, the ordering and delivery of the Veggie Meter device was substantially delayed due to the onset of the COVID-19 pandemic. Once the device became available, measurements were added to the protocol, and all subsequent participants were assessed without additional selection criteria. A comparison of baseline sociodemographic and medical history characteristics between those who underwent a skin carotenoid score measurement (n = 75) and those who did not (n = 65), yielded no significant differences suggesting limited potential for selection bias.

The Veggie Meter was calibrated prior to each test using the dark and white reference sticks provided by the manufacturer. Measurements were performed using the 3-scan mode according to the manufacturer instructions [44]. This includes having participants insert their index finger into the finger cradle of the Veggie Meter, with the fingertip pressed against the convex contact lens surface under the spring-loaded lid [44]. The pressure applied to the fingertip attenuates blood perfusion within the tissue of the fingertip, preventing blood from interfering with the measurements. Three scans were performed using the same finger and the Veggie Meter device calculates and displays the average of the three scores (range 0–800). This score was then recorded directly into the participant research chart. The skin carotenoid score from the Veggie Meter is a reliable and valid measurement, as the within-subject standard deviation over 55 repeated scans ranges between 3.4% and 4.1% [19].

*Dietary FV Intake.* Self-reported FV intake was obtained using the validated FV questionnaire (FVQ) [45,46]. The FVQ included five categories of consumption over seven consecutive days [45]. Categories were fruit juice, vegetable juice, fruit, potatoes (excluding French-fried potatoes), and vegetables (green salads and other vegetables). Servings of FV were estimated using standard analytic procedures and are presented as a daily value [45,46].

### 2.3. MetS Group Classification

MetS was defined according to the National Cholesterol Education Program (NCEP) Adult Treatment Panel (ATP) III, which was established based on a population from the United States [1,47,48]. Participants were considered to have MetS if they had three or more of the following components: (1) abdominal obesity (waist circumference > 102 cm in men and >88 cm in women), (2) elevated triglycerides (≥150 mg/dL or on drug treatment), (3) reduced HDL cholesterol (<40 mg/dL in men and <50 mg/dL in women or on drug treatment), (4) elevated blood pressure (≥130/85 mmHg or on anti-hypertensive medication), and (5) elevated fasting glucose (≥100 mg/dL or on drug treatment).

### 2.4. Statistical Analyses

Summary statistics were calculated, including means and standard deviations for continuous variables and frequencies with percentage (%) for categorical variables. The normality assumption for continuous variables was checked based on Shapiro–Wilk tests, and natural log-transformation was implemented if the assumption was violated. Unadjusted group comparisons between participants with MetS and without MetS were performed using two-sample *t*-tests or Wilcoxon rank-sum tests for continuous variables, and Pearson chi-square tests or Fisher exact tests for categorical variables, as appropriate. Spearman correlation coefficients were calculated to determine the association between total serum carotenoid levels and total skin carotenoid levels. For both aims, the relationships were further evaluated using multivariable models adjusting for demographic characteristics (age, sex, race and education) and other co-morbidities (coronary artery disease, cerebrovascular disease, peripheral artery disease, and chronic obstructive pulmonary disease). The *p*-values were obtained based on Wald tests and the partial R^2^ values quantifying the partial correlation of determination for each variable were calculated. All hypothesis tests were two-sided with the significance level of 0.05 and data were analyzed using SAS version 9.4 (Cary, NC, USA).

## 3. Results

**Figure 1:**
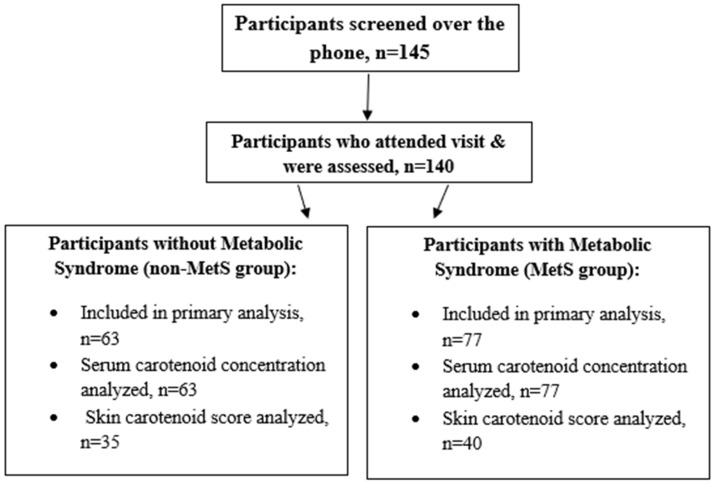
Participant flow and number analyzed by non-MetS and MetS groups.

Overall, 140 participants were included (n = 63 [45%] non-MetS group and n = 77 [55%] MetS group). Veggie Meter skin carotenoid scores were obtained on more than half of the sample (n = 35 non-MetS group and n = 40 MetS group). Participant flow through the study is detailed in Figure 1.

Participant characteristics by group are shown in Table 1. Compared to those without MetS, those with MetS had significantly higher values for body weight, body mass index, and body fat percentage than the control group. Furthermore, those with MetS were significantly more likely to be male, and to have other health conditions including coronary artery disease, hypertension, dyslipidemia, diabetes, and chronic obstructive pulmonary disease.

The serum carotenoid concentrations (ng/mL) and skin carotenoid scores in participants with and without MetS are shown in Table 2. The MetS group had a 52% lower serum alpha-carotene concentration (*p* < 0.001), a 39% lower serum beta-carotene concentration (*p* < 0.001), and a 22% lower total serum carotenoid concentration (*p* = 0.002) than the non-MetS group. There were no significant differences between groups for serum lycopene, serum lutein, serum cryptoxanthin, skin carotenoid score, or total FV intake. Group differences in total serum carotenoids were primarily driven by alpha- and beta-carotene levels. These results remained similar after adjusting for socio-demographic characteristics and other co-morbid conditions.

**Figure 2:**
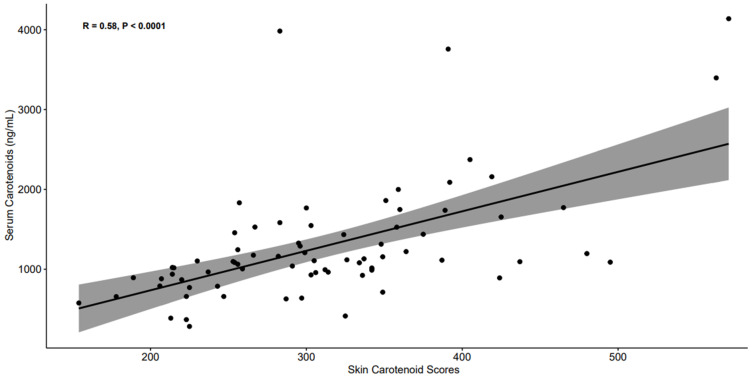
Scatter plot between the total serum carotenoid concentration and skin carotenoid scores.

To address the second aim of the study, serum and skin carotenoid values were first log-transformed because they were not normally distributed. There was a significant and positive association (r = 0.58, *p* < 0.001) between the total serum carotenoid concentration and skin carotenoid score (Figure 2).

The multivariable linear regression model evaluating the association between the total serum carotenoid concentration and the skin carotenoid score is shown in Table 3. The total serum carotenoid concentration was positively associated with the skin carotenoid score after adjusting for other covariates and co-morbid conditions (*p* < 0.001). There was a non-significant, positive correlation between total FV intake and serum carotenoids (r = 0.21, *p* = 0.07) and skin carotenoids (r = 0.19, *p* = 0.11) and these relationships remained non-significant after adjustment.

## 4. Discussion

There are two key findings in this study of older adults with and without MetS. First, total serum carotenoid concentration was 22% lower in the group with MetS as compared to the non-MetS group. This difference was primarily driven by alpha-carotene which was 52% lower, and beta-carotene which was 39% lower. Second, serum and skin carotenoid values were significantly, positively associated even after adjusting for other factors.

Although the unadjusted difference in total serum carotenoids between groups was statistically robust (*p* = 0.002), this association was attenuated after adjustment for demographic and other co-morbidities (*p* = 0.045). This attenuation suggests that some of the observed differences may be explained by confounding factors, that could be inherently linked to metabolic syndrome, the medical management of the condition, or carotenoid status. Similarly, while our multivariable model indicated a positive association between cerebrovascular disease and serum carotenoids, this finding should be interpreted with some caution given the small number of participants with this condition (n = 5). Taken together, these nuances highlight the complexity of disentangling carotenoid status from biological and behavioral influences on these concentrations in older adults with complex medical histories.

Overall, in general populations of adults with and without MetS, those with MetS have an 8–15% lower concentration of blood carotenoids [22,23,24,25]. Our results broadly agree with the other studies although we found a greater difference in total serum carotenoid concentration, as those with MetS were 22% lower [22,23,24,25]. This may be because the majority of previous reports focused on younger (aged 18+ years) or non-US populations, suggesting age-related physiological changes or cultural differences in dietary patterns could be contributing to the greater disparity observed in our older US sample [24].

While the present study included detailed medical histories, our brief dietary screener does not provide enough detail about dietary patterns to disentangle which of these factors, physiological changes or cultural differences in diet, may be most influential. In clinical settings, brief screeners are attractive because they are easy to administer, and this is why a brief screener was chosen for this study. However, by design they lack the granularity (i.e., food types, portion sizes, preparation methods) needed to accurately assess dietary intake for studies that involve biological processes. In fact, previous studies have demonstrated that they often show only weak associations with plasma carotenoids which may also be due to biological and behavioral variability of carotenoid transport and deposition [49]. There is a need for objective, low-burden alternatives for dietary assessment that can be integrated into clinical workflows. Spectroscopy-based skin carotenoids offer the potential of being an objective proxy for fruit and vegetable intake, providing a practical complement to comprehensive dietary methods [21,50,51,52]. This balance of feasibility and rigor is particularly relevant for routine clinical care and for studies in older adult populations, where respondent burden is a key consideration.

Only a few studies have examined carotenoid status exclusively in older adults with and without MetS and these were conducted in non-US samples [26,27]. For instance, in Chinese adults aged 50–75, compared to those without MetS, Liu et al. found that those with MetS had a 35% lower total carotenoid concentration, a 35% lower alpha carotene and 45% lower beta carotene concentration. While our estimates of total carotenoid concentration were slightly higher than that observed by Liu et al., the consistent pattern of lower total carotenoids, particularly lower alpha, and beta-carotene concentrations, among those with MetS reinforces the importance of these specific compounds as potential indicators of metabolic health. Data from a meta-analysis by Beydoun et al. also support this pattern and suggest that future studies should consider the relationship between MetS and lower alpha- and beta-carotene concentrations [24].

Our results are also similar to previous studies that have evaluated the association between serum and skin carotenoids [19,50,51,53]. In a study that recruited two separate convenience samples of adults in a retail setting (study 1) and a medical school (study 2), Jilcott Pitts et al. found that total serum carotenoid concentration was positively associated with skin carotenoid score (*p* = 0.001, r = 0.55) [50]. However, their study either did not assess medical history (study 1) or it excluded individuals with chronic disease (study 2), limiting generalizability to older adults with multimorbidity. In contrast, our sample included older adults with well-characterized medical histories, allowing us to explore the influence of comorbid conditions on this relationship. Notably, we observed that sex and a history of cerebrovascular disease contributed to variation in serum carotenoid levels suggesting that carotenoid metabolism may differ across sub-groups. In addition, age has been identified as a potential modifier of carotenoid status [54]. Although the mechanisms underlying these subgroup differences are unclear, they may reflect complex interactions between biological factors, comorbid conditions, and carotenoid metabolism that could warrant future investigation in future cohorts. To our knowledge, this is the first study to report on the association between RS-measured skin carotenoids and serum carotenoids in older adults with a well characterized medical history [51,52].

Although diet-related chronic disease is the leading cause of death and disability in the US and globally [10,55], accurate, feasible methods for assessing FV intake and nutritional biomarkers in routine clinical settings remain limited, particularly in older adults [12]. There is a critical need for low-cost, scalable approaches to assess nutritional status in this population. While further work is needed to disentangle the relationship between dietary intake, metabolic factors, and carotenoid status, our findings contribute to the growing body of evidence demonstrating that the RS skin carotenoid method (i.e., Veggie Meter) is a practical and valid alternative to serum carotenoid testing [19,50,51,53].

### Limitations and Strengths

There are several limitations associated with this study. Given the cross-sectional nature of the design, there may have been selection bias regarding study participation and results may not be generalizable to other populations. Significant differences found in the variables between the participants with and without MetS do not provide evidence of causality, even with statistical adjustment of covariates in multivariable models.

Furthermore, the physiological and metabolic differences between the MetS and non-MetS groups introduce inherent confounding that cannot be fully addressed through statistical adjustment. Individuals with MetS have greater metabolic dysregulation, chronic inflammation, and oxidative stress, all of which can independently influence carotenoid absorption, transport, metabolism, and tissue deposition [56,57]. These factors make it difficult to disentangle whether observed differences in serum carotenoid concentrations are attributable to dietary intake, altered metabolism, or both. As such, interpretation of these findings should be considered exploratory and hypothesis-generating.

Additionally, although our analyses were adjusted for covariates, it is possible that statistically significant findings are due to confounding variables that were not measured or residual confounding. Despite these limitations, statistical adjustment was necessary for our study design because of the difficulty in comparing participants in the MetS group, who have a relatively high burden of comorbidities and cardiovascular risk factors, with participants in the control group who have a lower burden.

Additionally, there are some limitations associated with the measurements of FV intake, skin carotenoids, and serum carotenoids. We did not find a significant association between diet and serum or skin carotenoids, and this is likely due to the use of a brief FV screener rather than a more detailed dietary assessment tool such as an FFQ or 24-h recall. Previous studies using FV screeners in both adults and children have had similar, non-significant results which, as noted above, suggests that FV screeners do not provide a detailed enough estimate of FV intake to correlate with serum or skin levels [58,59]. In addition, our measurement of skin carotenoid score utilized the Veggie Meter, which assesses total skin carotenoid level but does not provide measures of individual carotenoids as are measured in the serum. This may partially explain why the groups were not different by skin carotenoid score along with having fewer participants who were measured on the secondary outcome of skin carotenoids.

Over 92% of participants in our sample self-reported as white and data on sun exposure or skin pigmentation was not collected; all of which limits the generalizability of our findings regarding the skin carotenoid score. Skin pigmentation is a potential confounder for skin carotenoid measurements, and without adequate representation of individuals with varying levels of skin pigmentation, we cannot determine whether the observed serum–skin associations would hold across diverse populations. Future research should prioritize inclusion of racially and ethnically diverse participants with varying levels of skin pigmentation to ensure validity and equity in the application of this tool. While there has been some work to validate RS skin carotenoid methods in diverse racial and ethnic groups with a range of skin pigmentation [60,61], further validation in diverse groups is needed to understand the influence of racial, ethnic, and sun exposure-related skin pigmentation on this measurement.

## 5. Conclusions

Older adults with MetS had lower values of total serum carotenoids, alpha-carotene, and beta-carotene than participants without MetS. In addition, serum and skin carotenoid levels were positively correlated, even after adjusting for other confounding factors. These findings suggest that RS-measured skin carotenoid levels may offer a practical, non-invasive marker of carotenoid status in older adults. Given the ease of use and lower cost compared to serum measurement, this tool may be especially valuable in bridging the research-to-practice gap for routine assessment of carotenoid status in clinical settings serving older adult populations.

## Figures and Tables

**Table 1:** Clinical characteristics of participants with and without metabolic syndrome (MetS) ^a^.

Variables	Non-MetS Control Group (n = 63)	MetS Group (n = 77)	*p*-Value
Age (years)	68.0 ± 8.4	69.6 ± 7.7	0.278
Weight (kg)	69.3 ± 12.8	80.4 ± 14.8	<0.001
Height (cm)	164.8 ± 7.8	166.9 ± 10.4	0.346
Body Mass Index (kg/m^2^)	25.4 ± 3.9	28.8 ± 4.3	<0.001
Body Fat Percentage (%)	31.0 ± 7.5	34.5 ± 8.7	0.026
High-Sensitivity C-Reactive Protein, mg/L	1.8 ± 2.2	2.0 ± 2.0	0.316
Sex, men	15 (23.8)	38 (49.4)	0.003
Race, white	58 (92.1)	72 (93.5)	0.754
Education, college graduate	43 (68.3)	48 (62.3)	0.482
Coronary Artery Disease	2 (3.2)	17 (22.1)	<0.001
Cerebrovascular Disease	0 (0)	5 (6.5)	0.064
Peripheral Artery Disease	3 (4.8)	11 (14.3)	0.089
Chronic Kidney Disease	8 (12.7)	8 (10.4)	0.791
Peripheral Neuropathy	24 (38.1)	38 (49.4)	0.231
Current or Past Smoking	0 (0)	2 (2.6)	0.501
Hypertension	12 (19.0)	45 (58.4)	<0.001
Dyslipidemia	38 (60.3)	76 (98.7)	<0.001
Diabetes	0 (0)	9 (11.7)	0.004
Obesity	12 (19.0)	24 (31.2)	0.122
Arthritis	23 (36.5)	32 (41.6)	0.604
Chronic Obstructive Pulmonary Disease	2 (3.2)	11 (14.3)	0.038

^a^ Data are presented as means ± SD or n (%).

**Table 2:** Serum carotenoid concentrations, skin carotenoid score, and fruit and vegetable intake in participants with and without metabolic syndrome (MetS) ^a^.

Variables	Non-MetS Control Group (n = 63)	MetS Group (n = 77)	Unadjusted *p*-Value	Adjusted *p*-Value ^b^
Serum Alpha-Carotene, ng/mL	136.7 ± 143.6	66.0 ± 79.1	<0.001	0.001
Serum Beta-Carotene, ng/mL	491.2 ± 404.3	301.2 ± 371.9	<0.001	0.022
Serum Lycopene, ng/mL	470.1 ± 227.5	467.9 ± 326.2	0.289	0.693
Serum Lutein, ng/mL	183.8 ± 132.7	175.0 ± 130.7	0.443	0.851
Serum Cryptoxanthin, ng/mL	213.8 ± 298.8	160.3 ± 166.2	0.217	0.313
Total Serum Carotenoids, ng/mL	1495.6 ± 837.1	1170.5 ± 767.0	0.002	0.045
Skin Carotenoid Score ^c^	306.1 ± 76.5	317.3 ± 92.5	0.831	0.887
Fruit and Vegetable Intake, servings/day	5.1 ± 2.6	4.6 ± 1.8	0.678	0.420

^a^ Data are presented as means ± SD. ^b^ Adjusted for age, sex, race, education, coronary artery disease, cerebrovascular disease, peripheral artery disease, and chronic obstructive pulmonary disease. ^c^ Veggie Meter testing was conducted on a subset of participants, Non-MetS group n = 35 and MetS group n = 40.

**Table 3:** Multivariable linear regression model evaluating the association between total serum carotenoid concentration (ng/mL) and skin carotenoid score after adjusting for other covariates ^a^.

Multivariable Linear Regression Model	Estimate	95% CI	Partial R^2^ (%)	*p*-Value
Intercept	1.325	(−1.00, 3.653)		0.260
Skin Carotenoid Score	1.176	(0.820, 1.531)	40.5	<0.001
Metabolic Syndrome (MetS)	0.024	(−0.173, 0.220)	0.09	0.809
Age	−0.008	(−0.021, 0.005)	2.35	0.219
Sex, men	−0.254	(−0.458, −0.049)	8.76	0.016
Race, white	−0.291	(−0.737, 0.155)	2.59	0.197
Education, college graduate	−0.053	(−0.242, 0.136)	0.49	0.575
Coronary Artery Disease	−0.103	(−0.367, 0.161)	0.94	0.438
Cerebrovascular Disease	0.534	(0.038, 1.009)	6.76	0.028
Peripheral Artery Disease	0.073	(−0.315, 0.462)	0.22	0.709
Chronic Obstructive Pulmonary Disease	−0.218	(−0.553, 0.118)	2.56	0.199

^a^ Serum and skin carotenoid values were log transformed due to non-normal distribution.

## Data Availability

The data presented in this study are available on request from the corresponding author to mitigate risk of participant re-identification given the personal health information collected and the sample size.

## References

[1] Grundy S.M., Cleeman J.I., Daniels S.R., Donato K.A., Eckel R.H., Franklin B.A., Gordon D.J., Krauss R.M., Savage P.J., Smith S.C. (2005). Diagnosis and management of the metabolic syndrome: An American Heart Association/National Heart, Lung, and Blood Institute Scientific Statement. Circulation.

[2] O’Neill S., O’Driscoll L. (2014). Metabolic syndrome: A closer look at the growing epidemic and its associated pathologies. Obes. Rev..

[3] Lee J.-H., Lee K.-H., Kim H.-J., Youk H., Lee H.-Y. (2022). Effective Prevention and Management Tools for Metabolic Syndrome Based on Digital Health-Based Lifestyle Interventions Using Healthcare Devices. Diagnostics.

[4] Mozaffarian D., Hao T., Rimm E.B., Willett W.C., Hu F.B. (2011). Changes in diet and lifestyle and long-term weight gain in women and men. N. Engl. J. Med..

[5] Beltran-Sanchez H., Harhay M.O., Harhay M.M., McElligott S. (2013). Prevalence and trends of metabolic syndrome in the adult U.S. population, 1999–2010. J. Am. Coll. Cardiol..

[6] Mottillo S., Filion K.B., Genest J., Joseph L., Pilote L., Poirier P., Rinfret S., Schiffrin E.L., Eisenberg M.J. (2010). The metabolic syndrome and cardiovascular risk a systematic review and meta-analysis. J. Am. Coll. Cardiol..

[7] Ju S.Y., Lee J.Y., Kim D.H. (2017). Association of metabolic syndrome and its components with all-cause and cardiovascular mortality in the elderly: A meta-analysis of prospective cohort studies. Medicine.

[8] Guembe M.J., Fernandez-Lazaro C.I., Sayon-Orea C., Toledo E., Moreno-Iribas C. (2020). Risk for cardiovascular disease associated with metabolic syndrome and its components: A 13-year prospective study in the RIVANA cohort. Cardiovasc. Diabetol..

[9] Boudreau D., Malone D., Raebel M., Fishman P., Nichols G., Feldstein A., Boscoe A., Ben-Joseph R., Magid D., Okamoto L. (2009). Health care utilization and costs by metabolic syndrome risk factors. Metab. Syndr. Relat. Disord..

[10] Wang D.D., Li Y., Bhupathiraju S.N., Rosner B.A., Sun Q., Giovannucci E.L., Rimm E.B., Manson J.E., Willett W.C., Stampfer M.J. (2021). Fruit and Vegetable Intake and Mortality: Results From 2 Prospective Cohort Studies of US Men and Women and a Meta-Analysis of 26 Cohort Studies. Circulation.

[11] Wang X., Ouyang Y., Liu J., Zhu M., Zhao G., Bao W., Hu F.B. (2014). Fruit and vegetable consumption and mortality from all causes, cardiovascular disease, and cancer: Systematic review and dose-response meta-analysis of prospective cohort studies. BMJ.

[12] van Staveren W.A., de Groot L.C., Blauw Y.H., Van der Wielen R.P. (1994). Assessing diets of elderly people: Problems and approaches. Am. J. Clin. Nutr..

[13] Fukushima Y., Taguchi C., Kishimoto Y., Kondo K. (2023). Japanese carotenoid database with alpha- and beta-carotene, beta-cryptoxanthin, lutein, zeaxanthin, lycopene, and fucoxanthin and intake in adult women. Int. J. Vitam. Nutr. Res..

[14] Kim K., Madore M.P., Chun O.K. (2023). Changes in Intake and Major Food Sources of Carotenoids among U.S. Adults between 2009–2018. Metabolites.

[15] Rao A.V., Rao L.G. (2007). Carotenoids and human health. Pharmacol. Res..

[16] Dragsted L.O., Gao Q., Scalbert A., Vergères G., Kolehmainen M., Manach C., Brennan L., Afman L.A., Wishart D.S., Lacueva C.A. (2018). Validation of biomarkers of food intake—Critical assessment of candidate biomarkers. Genes. Nutr..

[17] Burrows T.L., Rollo M.E., Williams R., Wood L.G., Garg M.L., Jensen M., Collins C.E. (2017). A Systematic Review of Technology-Based Dietary Intake Assessment Validation Studies That Include Carotenoid Biomarkers. Nutrients.

[18] Pennant M., Steur M., Moore C., Butterworth A., Johnson L. (2015). Comparative validity of vitamin C and carotenoids as indicators of fruit and vegetable intake: A systematic review and meta-analysis of randomised controlled trials. Br. J. Nutr..

[19] Ermakov I.V., Ermakova M., Sharifzadeh M., Gorusupudi A., Farnsworth K., Bernstein P.S., Stookey J., Evans J., Arana T., Tao-Lew L. (2018). Optical assessment of skin carotenoid status as a biomarker of vegetable and fruit intake. Arch. Biochem. Biophys..

[20] Ermakov I.V., Gellermann W. (2012). Dermal carotenoid measurements via pressure mediated reflection spectroscopy. J. Biophotonics.

[21] Radtke M.D., Poe M., Stookey J., Pitts S.J., Moran N.E., Landry M.J., Rubin L.P., Stage V.C., Scherr R.E. (2021). Recommendations for the Use of the Veggie Meter^®^ for Spectroscopy-Based Skin Carotenoid Measurements in the Research Setting. Curr. Dev. Nutr..

[22] Ford E.S., Mokdad A.H., Giles W.H., Brown D.W. (2003). The metabolic syndrome and antioxidant concentrations: Findings from the Third National Health and Nutrition Examination Survey. Diabetes.

[23] Coyne T., Ibiebele T.I., Baade P.D., McClintock C.S., Shaw J.E. (2009). Metabolic syndrome and serum carotenoids: Findings of a cross-sectional study in Queensland, Australia. Br. J. Nutr..

[24] Beydoun M.A., Chen X., Jha K., Beydoun H.A., Zonderman A.B., Canas J.A. (2019). Carotenoids, vitamin A, and their association with the metabolic syndrome: A systematic review and meta-analysis. Nutr. Rev..

[25] Beydoun M.A., Shroff M.R., Chen X., Beydoun H.A., Wang Y., Zonderman A.B. (2011). Serum antioxidant status is associated with metabolic syndrome among U.S. adults in recent national surveys1–3. J. Nutr..

[26] Liu J., Shi W.-Q., Cao Y., He L.-P., Guan K., Ling W.H., Chen Y.-M. (2014). Higher serum carotenoid concentrations associated with a lower prevalence of the metabolic syndrome in middle-aged and elderly Chinese adults. Br. J. Nutr..

[27] Sugiura M., Nakamura M., Ogawa K., Ikoma Y., Yano M. (2015). High serum carotenoids associated with lower risk for the metabolic syndrome and its components among Japanese subjects: Mikkabi cohort study. Br. J. Nutr..

[28] May K., Pitts S.J., Stage V.C., Kelley C.J., Burkholder S., Fang X., Zeng A., Lazorick S. (2020). Use of the Veggie Meter^®^ as a tool to objectively approximate fruit and vegetable intake among youth for evaluation of preschool and school-based interventions. J. Hum. Nutr. Diet..

[29] Gardner A.W., Parker D.E., Montgomery P.S., Blevins S.M. (2014). Step-monitored home exercise improves ambulation, vascular function, and inflammation in symptomatic patients with peripheral artery disease: A randomized controlled trial. J. Am. Hear. Assoc..

[30] National Kidney Foundation (2002). K/DOQI clinical practice guidelines for chronic kidney disease: Evaluation, classification, and stratification. Am. J. Kidney Dis..

[31] Aboyans V., Criqui M.H., Abraham P., Allison M.A., Creager M.A., Diehm C., Fowkes F.G.R., Hiatt W.R., Jönsson B., Lacroix P. (2012). Measurement and interpretation of the ankle-brachial index: A scientific statement from the American Heart Association. Circulation.

[32] Expert Panel on Detection, Evaluation, and Treatment of High Blood Cholesterol in Adults (2001). Executive Summary of The Third Report of The National Cholesterol Education Program (NCEP) Expert Panel on Detection, Evaluation, and Treatment of High Blood Cholesterol In Adults (Adult Treatment Panel III). JAMA.

[33] Gardner A.W., Montgomery P.S., Casanegra A.I., Silva-Palacios F., Ungvari Z., Csiszar A. (2016). Association between gait characteristics and endothelial oxidative stress and inflammation in patients with symptomatic peripheral artery disease. Age.

[34] Zhang Q., Yi N., Liu S., Zheng H., Qiao X., Xiong Q., Liu X., Zhang S., Wen J., Ye H. (2018). Easier operation and similar power of 10 g monofilament test for screening diabetic peripheral neuropathy. J. Int. Med. Res..

[35] Armstrong D.G., Lavery L.A., Vela S.A., Quebedeaux T.L., Fleischli J.G. (1998). Choosing a practical screening instrument to identify patients at risk for diabetic foot ulceration. Arch. Intern. Med..

[36] Oyer D.S., Saxon D., Shah A. (2007). Quantitative assessment of diabetic peripheral neuropathy with use of the clanging tuning fork test. Endocr. Pract..

[37] Pietrobelli A., Rubiano F., St-Onge M.-P., Heymsfield S.B. (2004). New bioimpedance analysis system: Improved phenotyping with whole-body analysis. Eur. J. Clin. Nutr..

[38] Sluyter J.D., Schaaf D., Scragg R.K., Plank L.D. (2010). Prediction of fatness by standing 8-electrode bioimpedance: A multiethnic adolescent population. Obesity.

[39] Lee B.-L., New A.-L., Ong C.-N. (2003). Simultaneous determination of tocotrienols, tocopherols, retinol, and major carotenoids in human plasma. Clin. Chem..

[40] Kopec R.E., Schweiggert R.M., Riedl K.M., Carle R., Schwartz S.J. (2013). Comparison of high-performance liquid chromatography/tandem mass spectrometry and high-performance liquid chromatography/photo-diode array detection for the quantitation of carotenoids, retinyl esters, α-tocopherol and phylloquinone in chylomicron-rich fractions of human plasma. Rapid Commun. Mass. Spectrom..

[41] Colmán-Martínez M., Martínez-Huélamo M., Miralles E., Estruch R., Lamuela-Raventós R.M., Saso L. (2015). A New Method to Simultaneously Quantify the Antioxidants: Carotenes, Xanthophylls, and Vitamin A in Human Plasma. Oxidative Med. Cell. Longev..

[42] Vallverdú-Queralt A., Martínez-Huélamo M., Arranz-Martinez S., Miralles E., Lamuela-Raventós R.M. (2012). Differences in the carotenoid content of ketchups and gazpachos through HPLC/ESI(Li^+^)-MS/MS correlated with their antioxidant capacity. J. Sci. Food Agric..

[43] Hrvolová B., Martínez-Huélamo M., Colmán-Martínez M., Hurtado-Barroso S., Lamuela-Raventós R.M., Kalina J. (2016). Development of an Advanced HPLC–MS/MS Method for the Determination of Carotenoids and Fat-Soluble Vitamins in Human Plasma. Int. J. Mol. Sci..

[44] Obana A., Gohto Y., Gellermann W., Ermakov I.V., Sasano H., Seto T., Bernstein P.S. (2019). Skin Carotenoid Index in a large Japanese population sample. Sci. Rep..

[45] Godin G., Bélanger-Gravel A., Paradis A.-M., Vohl M.-C., Pérusse L. (2008). A simple method to assess fruit and vegetable intake among obese and non-obese individuals. Can. J. Public Health.

[46] Vézina-Im L.-A., Godin G., Couillard C., Perron J., Lemieux S., Robitaille J. (2016). Validity and reliability of a brief self-reported questionnaire assessing fruit and vegetable consumption among pregnant women. BMC Public Health.

[47] Huang P.L. (2009). A comprehensive definition for metabolic syndrome. Dis. Model. Mech..

[48] Zimmet P., Magliano D., Matsuzawa Y., Alberti G., Shaw J. (2005). The metabolic syndrome: A global public health problem and a new definition. J. Atheroscler. Thromb..

[49] Burrows T.L., Hutchesson M.J., Rollo M.E., Boggess M.M., Guest M., Collins C.E. (2015). Fruit and Vegetable Intake Assessed by Food Frequency Questionnaire and Plasma Carotenoids: A Validation Study in Adults. Nutrients.

[50] Pitts S.B.J., Jahns L., Wu Q., Moran N.E., Bell R.A., Truesdale K.P., Laska M.N. (2018). A non-invasive assessment of skin carotenoid status through reflection spectroscopy is a feasible, reliable and potentially valid measure of fruit and vegetable consumption in a diverse community sample. Public Health Nutr..

[51] Radtke M.D., Pitts S.J., Jahns L., Firnhaber G.C., Loofbourrow B.M., Zeng A., Scherr R.E. (2020). Criterion-Related Validity of Spectroscopy-Based Skin Carotenoid Measurements as a Proxy for Fruit and Vegetable Intake: A Systematic Review. Adv. Nutr..

[52] Jilcott Pitts S.B., Johnson N.S., Wu Q., Firnhaber G.C., Preet Kaur A., Obasohan J. (2022). A meta-analysis of studies examining associations between resonance Raman spectroscopy-assessed skin carotenoids and plasma carotenoids among adults and children. Nutr. Rev..

[53] Pitts S.J., Moran N.E., Laska M.N., Wu Q., Harnack L., Moe S., Carr-Manthe P., Gates E., Chang J., Zaidi Y. (2023). Reflection Spectroscopy-Assessed Skin Carotenoids Are Sensitive to Change in Carotenoid Intake in a 6-Week Randomized Controlled Feeding Trial in a Racially/Ethnically Diverse Sample. J. Nutr..

[54] Pitts S.B.J., Wu Q., Moran N.E., Laska M.N., Harnack L. (2023). Examining Potential Modifiers of Human Skin and Plasma Carotenoid Responses in a Randomized Trial of a Carotenoid-Containing Juice Intervention. J. Nutr..

[55] Collaborators G.B.D.D. (2019). Health effects of dietary risks in 195 countries, 1990–2017: A systematic analysis for the Global Burden of Disease Study 2017. Lancet.

[56] Harari A., Coster A.C.F., Jenkins A., Xu A., Greenfield J.R., Harats D., Shaish A., Samocha-Bonet D. (2019). Obesity and Insulin Resistance Are Inversely Associated with Serum and Adipose Tissue Carotenoid Concentrations in Adults. J. Nutr..

[57] Masenga S.K., Kabwe L.S., Chakulya M., Kirabo A. (2023). Mechanisms of Oxidative Stress in Metabolic Syndrome. Int. J. Mol. Sci..

[58] Morgan E.H., Graham M.L., Marshall G.A., Hanson K.L., Seguin-Fowler R.A. (2019). Serum carotenoids are strongly associated with dermal carotenoids but not self-reported fruit and vegetable intake among overweight and obese women. Int. J. Behav. Nutr. Phys. Act..

[59] Seguin-Fowler R.A., Hanson K.L., Marshall G.A., Belarmino E.H., Pitts S.B.J., Kolodinsky J., Sitaker M., Ammerman A. (2021). Fruit and Vegetable Intake Assessed by Repeat 24 h Recalls, but Not by A Dietary Screener, Is Associated with Skin Carotenoid Measurements in Children. Nutrients.

[60] Di Noia J., Gellermann W. (2021). Use of the Spectroscopy-Based Veggie Meter^®^ to Objectively Assess Fruit and Vegetable Intake in Low-Income Adults. Nutrients.

[61] Nagao-Sato S., Baltaci A., Reyes A.O.P., Zhang Y., Choque G.A.H., Reicks M. (2021). Skin Carotenoid Scores Assessed with Reflection Spectroscopy Are Associated with Self-Reported Fruit and Vegetable Intake Among Latino Early Adolescents. J. Acad. Nutr. Diet..

